# Proteomic Analysis of Urine from Patients with *Plasmodium vivax* Malaria Unravels a Unique *Plasmodium vivax* Protein That Is Absent from *Plasmodium falciparum*

**DOI:** 10.3390/tropicalmed7100314

**Published:** 2022-10-18

**Authors:** Raianna F. Fantin, Claudia Abeijon, Dhelio B. Pereira, Ricardo T. Fujiwara, Lilian L. Bueno, Antonio Campos-Neto

**Affiliations:** 1 Department of Parasitology, Federal University of Minas Gerais, Belo Horizonte 31270-901, MG, Brazil; 2 DetectoGen Inc., Westborough, MA 01581, USA; 3 Centro de Pesquisas em Medicina Tropical de Rondônia (CEPEM), Porto Velho 76812-329, RO, Brazil; 4 Department of Infectious Disease and Global Health, Cummings School of Veterinary Medicine, Tufts University, North Grafton, MA 01536, USA

**Keywords:** rapid antigen detection test, malaria, *Plasmodium vivax*, *Plasmodium falciparum*, Vir14

## Abstract

Five species of *Plasmodium* cause malaria in humans and two of them, *P. vivax* and *P. falciparum*, pose the greatest threat. Rapid antigen detection tests (RADT) have been used for many years to diagnose and distinguish malaria caused by these two parasites. *P. falciparum* malaria can single-handedly be diagnosed using an RADT, which detects the unique *P. falciparum* specific histidine-rich protein 2 (HRP2). Unfortunately, there is no RADT that can single-handedly diagnose *P. vivax* malaria because no specific marker of this parasite has yet been described. Here, we report the discovery of a unique *P. vivax* protein (Vir14, NCBI Reference Sequence: XP_001612449.1) that has no sequence similarity with proteins of *P. falciparum* and no significant similarities with proteins of other species of *Plasmodium*. We propose that this protein could be an outstanding candidate molecule for the development of a promising RADT that can single-handedly and specifically diagnose *P. vivax* malaria.

## 1. Introduction

The World Health Organization (WHO) projected that the worldwide incidence of malaria in 2020 was 241 million cases and 627,000 deaths (https://www.who.int/news-room/fact-sheets/detail/malaria, accessed on 26 July 2022). Five species of *Plasmodium* cause malaria in humans, namely *P. falciparum*, *P. vivax*, *P. malariae*, *P. ovale* and *P. knowlesi*. Two of them, *P. vivax* and *P. falciparum*, pose the greatest threat to humans [1]. The reference standard diagnosis of malaria is the microscopic observation of the parasites in stained blood smears [2]. PCR tests are relatively more sensitive and precise than microscopy [3,4]. However, nucleic acid-based tests remain complicated and expensive for routine use in malaria endemic areas. Conventional serological tests are of limited value because, in endemic areas, most people have antibody titers from previous malaria infections [5]. Rapid antigen detection tests (RADT) have been used for many years [6] and the WHO recommends that all cases of suspected malaria be confirmed by either gold standard microscopy or RADT before the administration of treatment (https://www.cdc.gov/malaria/diagnosis_treatment/clinicians1.html, accessed on 30 September 2022).

The currently available RADTs primarily use two *Plasmodium* antigens [6,7,8]. One is the *P. falciparum* specific histidine-rich protein 2 (HRP2) and the second is the pan-malarial antigen lactate dehydrogenase (LDH). *Plasmodium* aldolase, which is also a pan-malarial antigen, has been used in some RADTs [9]. HRP2-based RADT is used to diagnose *P. falciparum* malaria single-handedly and specifically.

These RADTs have unquestionably facilitated the diagnosis of malaria, however the tests have limitations, such as: 1. variability in their sensitivity/specificity; 2. of the three markers used in the tests, only HRP2 is a species-specific marker (*P. falciparum*); and 3. there is no specific RADT for *P. vivax* malaria because no specific marker of this parasite has been described.

We have recently used mass spectroscopy to successfully identify *Plasmodium* protein biomarker candidates that are excreted in the urine of malaria patients. One of the identified proteins (variable surface protein Vir14, NCBI Reference Sequence: XP_001612449.1) is of great interest in that it is highly unique to *P. vivax*, with no significant similarity with any protein of *P. falciparum*. Vir14 also lacks homology with proteins of *P. malariae*, *P. ovale*, and *P. knowlesi*, as well as with any human proteins.

## 2. Material and Methods

### 2.1. Clinical Specimens

Four stored frozen and de-identified urine samples and 121 serum samples were from *P. vivax* malaria patients from the Amazon area in Brazil were obtained. The patients were from the Research Center for Tropical Medicine (CEPEM), Porto Velho, RO. Approval to use these samples was obtained from the Human Research Ethics Committee—COEP (CAAE-00842112.2.0000.5149).

### 2.2. Mass Spectroscopy Analysis

**Figure 1:**
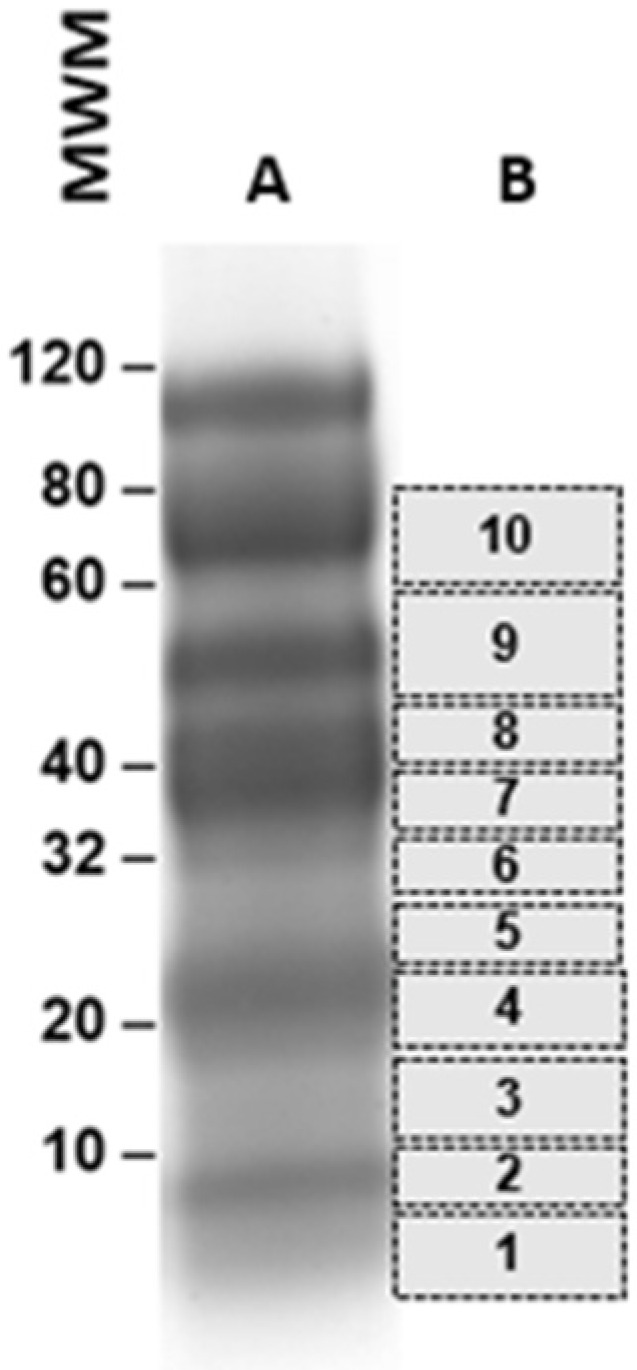
Illustration of gel bands that were cut from PAGE for subsequent mass spectroscopy analysis. The patient urine samples (3 mL) had a concentration of ~300 µL and were subjected to PAGE, followed by Coomassie blue staining (**A**). Ten bands were cut from the gel (**B**) and subjected to mass spectroscopy for the identification of *P. vivax* peptide sequences.

Individual urine samples (3–4 mL) were concentrated using Centricon P3 (3 kDa cutoff filters) to ~200–300 μL. Equal volumes of concentrated urine samples were mixed with an electrophoresis sample buffer and then submitted to SDS-PAGE, followed by Coomassie staining. Bands ranging from ~5 kDa to ~75 kDa were excised from the gel (Figure 1) and submitted for mass spectroscopy (MS) analysis at the Taplin Mass Spectrometry Facility, Harvard Medical School, Boston, MA. For each urine sample, eight to ten bands were cut from the gel. Each band was then independently submitted to MS runs. Gel bands were trypsin-digested into peptides. Peptides were analyzed by nano-scale liquid chromatography coupled to a tandem mass spectrometer. Eluted peptides first had their molecular masses measured and were then fragmented before the fragment masses were measured. The specific fragmentation pattern was computer-searched against predicted tryptic peptides from all known proteins from genome sequencing projects of humans and *Plasmodium* parasites. The power of this technique is in its redundancy. As many peptides are generated from the initial gel band, multiple matches to the protein of interest were detected. In this way, the protein identity is completely unambiguous. Peptide score cutoff values were chosen at Xcorr of >1.8 for singly charged ions, 2.5 for double charged ions, and 3.0 for triple charged ions, along with deltaCN values of 0.1, and RSP values of 1. The cross-correlation values chosen for each peptide assured a high confidence match for the different charge states, while the deltaCN cutoff ensured the uniqueness of the peptide hit. The RSP value of 1 ensured that the peptide matched the top hit in the preliminary scoring and that the peptide fragment file only matched one protein hit.

### 2.3. Recombinant Protein and ELISA

Expression and purification of recombinant Vir14 protein and direct ELISA were performed, as previously published [10,11,12].

## 3. Results

### 3.1. Discovery and Characterization of a Unique P. vivax Protein Present in the Urine of Patients with P. vivax Malaria from Brazil

**Figure 2:**
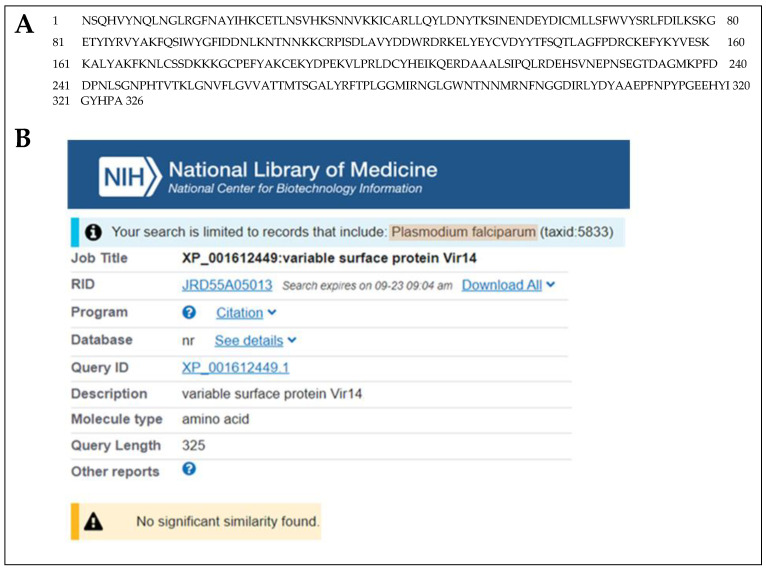
Amino acid sequence and NCBI BLAST analysis (protein/protein) of *P. vivax* Vir14 (XP_001612449.1). The BLAST analysis of the full-length protein sequence (**A**) revealed that the Vir14 protein is unique to *P. vivax* and that it has no significant similarity with any proteins of *P. falciparum* proteins (**B**), proteins of other *Plasmodium* species (not shown) or with human proteins (not shown).

For the malarial antigen discovery strategy, we used the same protocol that we previously and successfully employed for the identification of *Leishmania infantum*/*Leishmania donovani* proteins in the urine of visceral leishmaniasis (VL) patients [10,11,12,13,14]. For the current study, we used urine samples from malaria patients from the Amazon area in Brazil. The patients were from the Centro de Pesquisas em Medicina Tropical de Rondônia (CEPEM), Porto Velho, RO. The samples were collected before the initiation of therapy and were from patients diagnosed with malaria, based on the following criteria: (a) a clinical course consistent with malaria, including a fever, chills, headaches, muscular aching and weakness, vomiting, coughing, diarrhea and abdominal pain; and (b) laboratory findings that confirm the presence of *P. vivax* by both thick Giemsa stain blood smear microscopy and PCR [15,16,17]. Parasitemia levels were 500–10,000/µL of blood. None of the patients had any clinical symptoms or laboratory findings compatible with renal or urinary tract abnormalities, and none of them were receiving anti malaria therapy at the time of urine collection. Urine samples were collected from four *P. vivax* malaria patients. Urine was concentrated using Centricon <3 kDa and submitted to PAGE Coomassie blue stain. Bands were excised from the gels and submitted for mass spectroscopy analysis (MS), which revealed that all four urine samples contained at least one putative *P. vivax* protein (Table 1). These proteins were unambiguously identified because the criteria used in the MS to define the significance of a peptide “hit” were above the expected rankings, i.e., XCorr >2.0 and DelCn >0.1 [18,19]. Among the five identified putative *P. vivax* proteins listed in Table 1, the approach revealed the *P. vivax* variable surface protein Vir14 (highlighted in green, NCBI Reference Sequence: XP_001612449.1), which was present in the urine of two of the four *P. vivax* patients analyzed (band #8, Figure 1). The BLAST analysis of Vir14 revealed no significant similarity with any protein of *P. falciparum*, even when this organism was specifically included in the search (Figure 2). The other four identified proteins were interpreted to be of lesser interest for future RADT development specific for *P. vivax* malaria because they had significant similarities with proteins of *P. falciparum*. Consequently, these observations confirmed that Vir14 is a unique candidate molecule for the development of an RADT highly specific for *P. vivax*.

### 3.2. Recognition of Vir14 by Sera from Patients with P. vivax Malaria

To begin the validation of Vir14 as a *P. vivax* marker produced in vivo during infection, we initially investigated the presence of anti-Vir14 specific antibodies in the sera of malaria patients (Table 2). It is important to emphasize that this approach did not aim to evaluate the usefulness of Vir14 as a target antigen for the serological diagnosis of *P. vivax* malaria. The aim was to indirectly confirm that Vir14 is indeed an antigen that is produced by the parasite during the disease, thus an antigen that is suitable as target for the development of an antigen detection test. Sera were collected from *P. vivax*-infected subjects (n = 121) and from healthy control subjects (n = 6) from Brazil). This was assessed for in relation to the antibody recognition of Vir14. This assessment was carried out by direct antibody ELISA using purified recombinant Vir14 as antigen and sera diluted at 1/50. We found that 61% of patients presented circulating antibodies against Vir14. These findings strongly suggest that Vir14 is a *P. vivax* protein that is actively produced in vivo during the disease.

## 4. Discussion

*P. falciparum* malaria can single-handedly be diagnosed using an RADT that detects the unique *P. falciparum* specific histidine-rich protein 2 (HRP2). Unfortunately, there is currently no RADT that can single-handedly be used for the of *P. vivax* malaria. To diagnose this type of malaria, the existing RADTs are formatted to detect both the pan-malarial lactate dehydrogenase (LDH) antigen as well the *P. falciparum* specific HRP2 molecule in order to exclude the possibility of *P. falciparum* malaria. A positive result for both HRP2 and LDH assures the diagnosis of *P. falciparum* malaria. A negative HRP2 result and a positive LDH excludes the diagnosis of *P. falciparum* malaria, but does not specify which other malaria is being diagnosed because LDH is a ubiquitous pan-malarial antigen. This limitation unquestionably creates a diagnostic complication that unfortunately occurs in areas of the world (e.g., South America) where malaria is caused by *P. vivax* and by other *Plasmodium* species, including *P. falciparum*. In these co-endemic malarial areas, this diagnostic distinction is imperative because of the need to separate the treatment approach for *P. falciparum* and *P. vivax* [20,21,22,23]. Further, the CDC guidelines to treat malaria emphasizes that determination of the infecting *Plasmodium* species for treatment purposes is critical for four main reasons: first, *P. falciparum* can cause rapidly progressive severe illness or death, while *P. vivax* is less likely to cause severe disease. Second, *P. vivax* infections also require treatment for the hypnozoites, which remain dormant in the liver and can cause relapsing episodes. Third, *P. falciparum* and *P. vivax* species have different drug resistance patterns in different geographic regions of the world. Finally, for *P. falciparum*, the urgent initiation of appropriate therapy is especially critical (https://www.cdc.gov/malaria/diagnosis_treatment/clinicians1.html, accessed on 30 September 2022). Therefore, an RADT that can specifically and single-handedly diagnose *P. vivax* malaria will be of great significance [24].

We have previously described an interesting alternative approach for the direct identification of *Mycobacterium tuberculosis* and *Leishmania* antigens in urine samples of patients with tuberculosis and visceral leishmaniasis [10,11,12,25,26,27]. We performed mass spectroscopy (MS) analysis in urine samples instead of blood because urine has far less host proteins than blood, thus enormously diminishing the background results generated by the MS. In addition, the rationale of using urine as a source of the pathogen’s antigens was based on the premise that microbial proteins or their breakdown products (peptides) produced in vivo, e.g., in organs like liver, lung, bone marrow, blood, etc., will have to be eliminated from the body, which will be physiologically and most likely be excretion in the urine. The translation of our original strategy of antigen discovery to patients with *P. vivax* malaria was readily achieved. Using this approach, we were able to discover a unique protein from this parasite that could be of outstanding interest for the development of an RADT that can single-handedly and specifically diagnose *P. vivax* malaria.

The discovered protein, variable surface protein Vir14, which is putative (Vir14) from *P. vivax*, has no significant similarity with any protein of *P. falciparum.* In addition, the protein has little similarity with proteins of *P. malariae*, *P. ovale*, and *P. knowlesi.* NCBI protein-protein BLAST of *P. vivax* Vir14 using databases (taxid) of these *Plasmodium* shows similarities of only 27%, 36% and 20% with proteins of these parasites, respectively. No significant similarity exists between Vir14 and human proteins. Moreover, using conventional ELISA, we have found that 61% of patients with *P. vivax* malaria have anti-Vir14 antibodies. Finally, Vir14 is produced by *P. vivax* merozoites and is exported from the cytoplasm of the parasites to the membrane of infected red blood cells [28].

While Vir14 expresses some areas of amino acid sequence variability among variants of *P. vivax*, it nonetheless has extensive areas of conserved sequences in over 100 sequences of this *Plasmodium* species, as revealed by the NCBI BLAST analysis. It is important to emphasize that these are species-conserved sequences, i.e., they are unique to *P. vivax*, and are only minorly present in other species of *Plasmodium* and absent in *P. falciparum.* Therefore, a highly sensitivity and specific *P. vivax* RADT can be successfully developed using monoclonal antibodies specific for the conserved sequences of Vir14.

The fact that Vir14 was initially found by MS in two out of the four urine samples from *P. vivax* malaria, means, in our view, that this small sample size should not be considered not significant for the translation of this finding to a *P. vivax* specific RADT. What is relevant was the serendipitous unraveling of the undisputed fact that *P. vivax* produces a protein that is unique to this species of *Plasmodium* and that this protein is completely absent in *P. falciparum.* It is also important to mention the fact that Vir14 is a protein that is produced by all *P. vivax* thus far sequenced (NCIB BLAST analysis not shown). Therefore, an antigen detection test that uses blood samples should be successful, because, by definition, the parasite (and Vir14) is present in 100% of patients with the disease.

In conclusion, we have unraveled a unique protein of *P. vivax* that has an enormous potential for the development of an RADT that can single-handedly diagnose *P. vivax* malaria. This suggestion is supported by the following premises: 1. Vir14 was found in bodily fluids of patients with *P. vivax* malaria; 2. Vir14 is a unique protein of *P. vivax* that is absent in *P. falciparum* and other *Plasmodium* species; 3. Vir14 is produced by *P. vivax* merozoites and has been detected in the membrane of infected human red blood cells; and 4. The Vir14 protein is actively produced in vivo during disease, as patients with *P. vivax* malaria have anti-Vir14 antibodies in their blood. Nonetheless, it is important to keep in mind that, for most RADTs, the concentration of the marker in the patient’s sample is a critical condition for the success of this diagnostic strategy. Presently, there is no information about the concentration of Vir14 in the blood of patients with *P. vivax* malaria.

Finally, in collaboration with the company Safetest, Belo Horizonte, MG, Brazil, we are currently working on the development of a Vir14-based RADT for the diagnosis of *P. vivax* malaria.

## Figures and Tables

**Table 1:** *Plasmodium vivax* peptides identified by mass spectroscopy in urine samples of patients with *P. vivax* malaria from Brazil.

Peptide Identified in Patient Urines	XCorr	ΔCorr	*P. vivax* Donor Protein	MW (Da)	Identity with *P. falciparum* Protein	Accession Numbers *P. vivax* (*Pv*) *P. falciparum* (*Pf*)
KMNLDEFNELVEQRNR	2.48	0.125	Proteasome regulatory subunit p27	33,710.41	>75%	XP_001614534.1 *Pv* XP_001351248.1 *Pf*
TINEGQTLLTVFK YQFINIER	3.46 1.88	0.623 0.160	Profilin	19,287.32	>95%	XP_001608363.1 *Pv* XP_001352188.1 *Pf*
GVDMHNEEIKAVIK	2.46	0.205	Uncharacterized protein	44,559.84	50%	XP_001614264.1 *Pv* XP_001350199.1 *Pf*
DAAALSIPQLR	2.51	0.248	Variable surface protein Vir14	37,719.38	**None**	XP_001612449.1 *Pv*
EELNKINYNPR	2.63	0.351	Heat shock protein, class I	26,949.43	>58%	XP_001616584.1 *Pv* XP_001350519.1 *Pf*

**Table 2:** Detection of anti-Vir14 specific antibodies in the sera of P. vivax malaria patients.

	Normal Healthy Control Subjects (6)	*P. vivax* Malaria Patients (n = 121)
Positive sera (% of patients) *	0	61

(*) Sera were diluted at 1/50 based on previous titration using sera from normal healthy control subjects. The cutoff OD to consider a serum from *P. vivax* malaria patient positive was calculated as the mean of the OD obtained for the sera from the normal healthy control subjects plus 3 standard deviations of that mean.

## Data Availability

The data presented in this study are available on request from the corresponding authors.
